# Supplementary material for: Generative artificial intelligence and machine learning methods to screen social media content
Source: PeerJ Comput Sci. 2025 Mar 14;11:e2710. doi: 10.7717/peerj-cs.2710 (PMC11935761; doi:10.7717/peerj-cs.2710)
Supplement: Supplemental Information 2 — To collect these hashtag pairs, we identified six broad keywords related to both pregnancy and vaping (“e-cigarette pregnant,” “e-cigarette pregnancy,” “vaping pregnant,” “vaping pregnancy,” “vape pregnant,” “vape pregnancy”) and used a scraper to collect metadata. We obtained 1,561 TikTok videos and, after removing duplicates, reviewed 600 videos, resulting in 171 eligible videos discussing both pregnancy and vaping. We extracted hashtags from metadata descriptions, applied stopword removal and the Porter Stemmer, and performed frequency analysis to identify the most common hashtags and pairs, as well as frequent words in post descriptions. By visualizing the co-occurrence network of hashtags, we identified 70 thematic hashtag pairs. [file peerj-cs-11-2710-s002.docx]

| **HashTag 1 related to pregnancy** | **Hashtag 2 related to vaping** | **Number of Unique posts** |
| --- | --- | --- |
| pregnancy | vape | 308 |
| Pregnancytiktok | vape | 307 |
| Bumpdate | vape | 322 |
| Pregnanttiktok | vape | 306 |
| Momsunder25 | vape | 300 |
| Momsoftiktok | vape | 307 |
| Pregnancytok | vape | 325 |
| Momtok | vape | 293 |
| Pregnantlife | vape | 304 |
| Expectingmom | vape | 288 |
| motherhood | vape | 345 |
| babybump | vape | 306 |
| pregnancy | ecigarette | 363 |
| Pregnancytiktok | ecigarette | 365 |
| Bumpdate | ecigarette | 356 |
| Pregnantiktok | ecigarette | 348 |
| Momsunder25 | ecigarette | 366 |
| Momsoftiktok | ecigarette | 377 |
| Pregnancytok | ecigarette | 362 |
| Momtok | ecigarette | 354 |
| Pregnantlife | ecigarette | 357 |
| Expectingmom | ecigarette | 367 |
| motherhood | ecigarette | 350 |
| babybump | ecigarette | 361 |
| Pregnancytiktok | vaping | 332 |
| pregnancy | quitsmoking | 331 |
| Bumpdate | quitsmoking | 347 |
| Momsunder25 | quitsmoking | 351 |
| Momsoftiktok | quitsmoking | 359 |
| Pregnancytok | quitsmoking | 344 |
| Momtok | quitsmoking | 338 |
| Pregnantlife | quitsmoking | 318 |
| Expectingmom | quitsmoking | 332 |
| motherhood | quitsmoking | 349 |
| babybump | quitsmoking | 326 |
| pregnanytiktok | quitsmoking | 318 |
| pregnancy | smoke | 334 |
| Bumpdate | smoke | 368 |
| Momsunder25 | smoke | 320 |
| Momsoftiktok | smoke | 321 |
| Pregnancytok | smoke | 337 |
| Momtok | smoke | 306 |
| Pregnantlife | smoke | 324 |
| Expectingmom | smoke | 318 |
| motherhood | smoke | 303 |
| babybump | smoke | 304 |
| pregnanytiktok | smoke | 347 |
| pregnancy | stopsmoking | 331 |
| Bumpdate | stopsmoking | 342 |
| Momsunder25 | stopsmoking | 347 |
| Momsoftiktok | stopsmoking | 355 |
| Pregnancytok | stopsmoking | 336 |
| Momtok | stopsmoking | 356 |
| Pregnantlife | stopsmoking | 325 |
| Expectingmom | stopsmoking | 321 |
| motherhood | stopsmoking | 343 |
| babybump | stopsmoking | 331 |
| pregnanytiktok | stopsmoking | 331 |
| pregnancy | juul | 351 |
| Pregnancytiktok | juul | 325 |
| Bumpdate | juul | 354 |
| Pregnanttiktok | juul | 343 |
| Momsunder25 | juul | 335 |
| Momsoftiktok | juul | 354 |
| Pregnancytok | juul | 341 |
| Momtok | juul | 362 |
| Pregnantlife | juul | 376 |
| Expectingmom | juul | 315 |
| motherhood | juul | 376 |
| babybump | juul | 363 |
| thirdtrimest | vape | 311 |
