# Supplementary material for: Generative artificial intelligence and machine learning methods to screen social media content
Source: PeerJ Comput Sci. 2025 Mar 14;11:e2710. doi: 10.7717/peerj-cs.2710 (PMC11935761; doi:10.7717/peerj-cs.2710)
Supplement: Supplemental Information 3 [file peerj-cs-11-2710-s003.docx]

## Appendix B.

**Supplemental Figure B1:** Top 20 Objects Detected in Video Frames Focusing on Vaping and Pregnancy

**[Supplemental Figure B1]**

**Supplemental Figure B2:** Top 20 Words Detected in Video Frames Focusing on Vaping and Pregnancy

**[Supplemental Figure B2]**
