# Supplementary figures and images for: Generative artificial intelligence and machine learning methods to screen social media content
Source: PeerJ Comput Sci. 2025 Mar 14;11:e2710. doi: 10.7717/peerj-cs.2710 (PMC11935761; doi:10.7717/peerj-cs.2710)

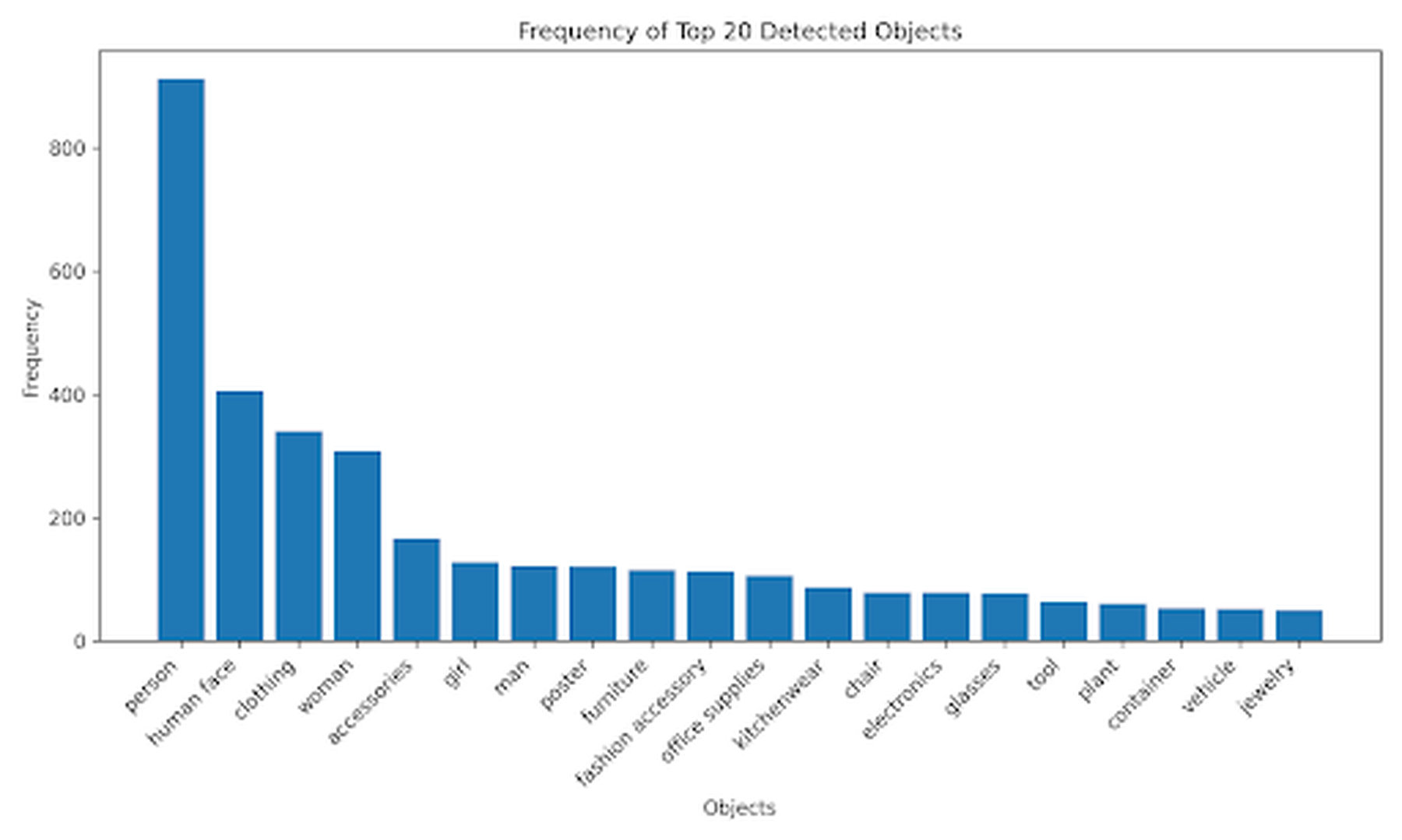

Supplement: Supplemental Information 4 [file peerj-cs-11-2710-s004.png]

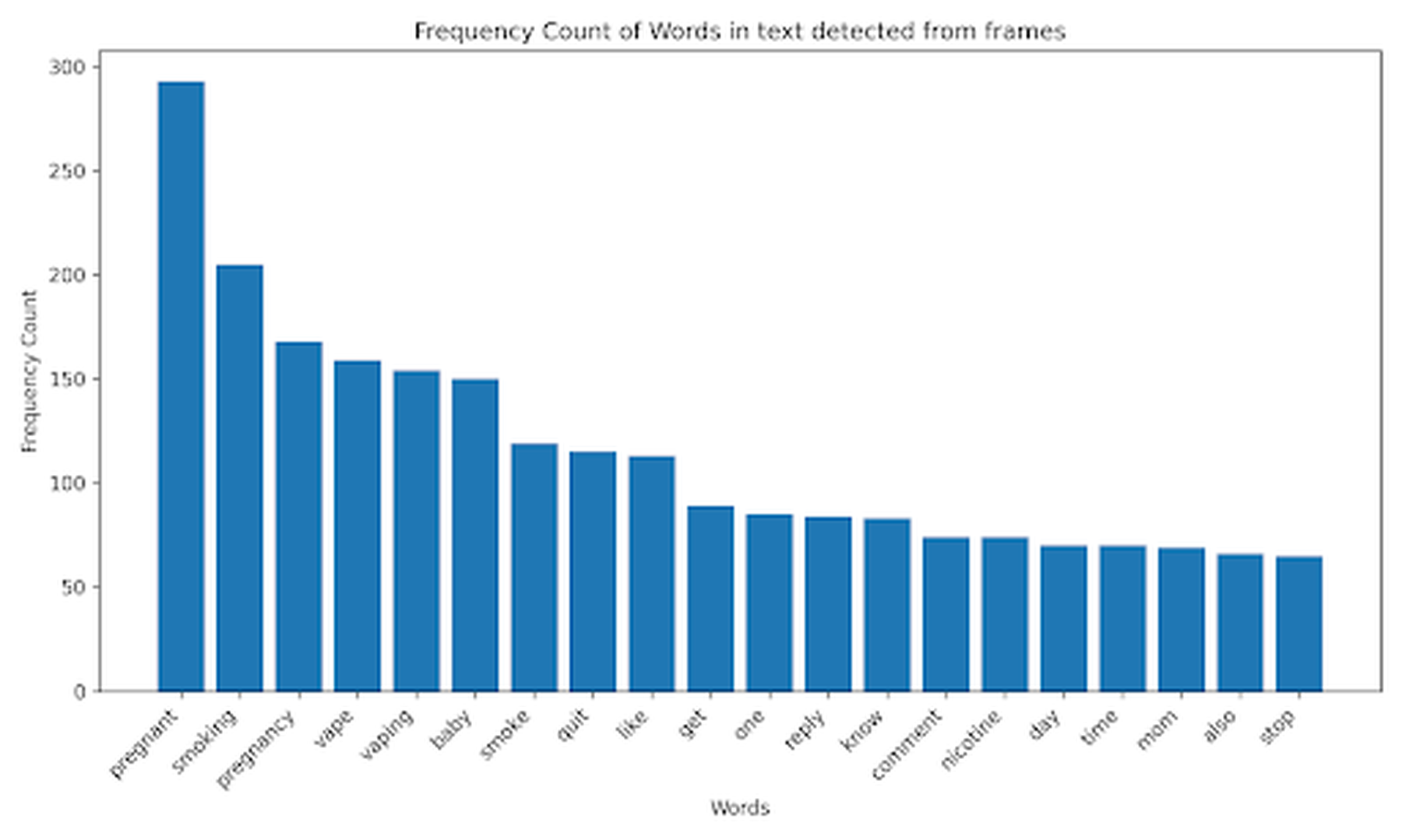

Supplement: Supplemental Information 5 [file peerj-cs-11-2710-s005.png]
